# Congenital Arhinia: A Rare Case

**Published:** 2014-01-01

**Authors:** Zhang Mao-Mao, Hu Yang-Hong, He Wei, Hu Kui-Kui

**Affiliations:** 1 Department of Plastic Surgery, Guangdong Women and Children's Hospital, Guangzhou Medical University, Guangzhou, 510010, China; 2 Department of Plastic Surgery, Second Affiliated Hospital to Nanchang University, Nanchang, 330006, China

**Dear Sir,**

The lack of external nose is generally a part of the complex malformation characterized by the absence of the nasal cavities, microphthalmia or coloboma of olfactory bulbs, high arched palate, coloboma of iris, and microtia. Only 43 cases of arhinia have been reported to date [1-4].

A healthy 40-year-old, gravida 5 and para 5, woman delivered a male infant weighing 2.89 kg by spontaneous vaginal delivery at term. There was neither a family background of congenital malformations nor a history of any medications during the pregnancy. The prenatal course was uncomplicated. On examination, the baby showed absence of the nose, nasal root, nasal pits; microphthalmia, auris dextra microtia, right side anorchia, small penis, high arched palate, hypoplasia of bilateral maxillary sinus and ethmoid sinus (Fig. 1-3). There were longitudinal ridge-like protuberances in the mid-face. Shortly, the infant was transferred to the neonatal intensive care unit for further evaluation and monitoring. The baby had severe shortness of breath which was relieved by an oropharyngeal tube. Karyotyping performed was 46 XY. The dyspnea and tachypnea greatly improved one month later and the baby adapted to oral breathing and feeding simultaneously. Further work-up including echocardiogram, CT brain, and blood chemistry, all were normal.

**Figure:**
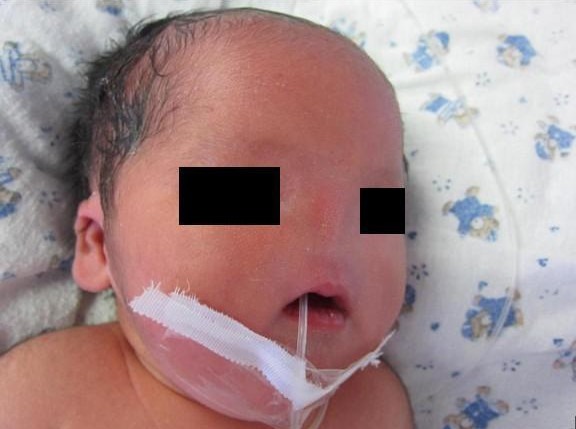
Figure 1: Clinical photograph

**Figure:**
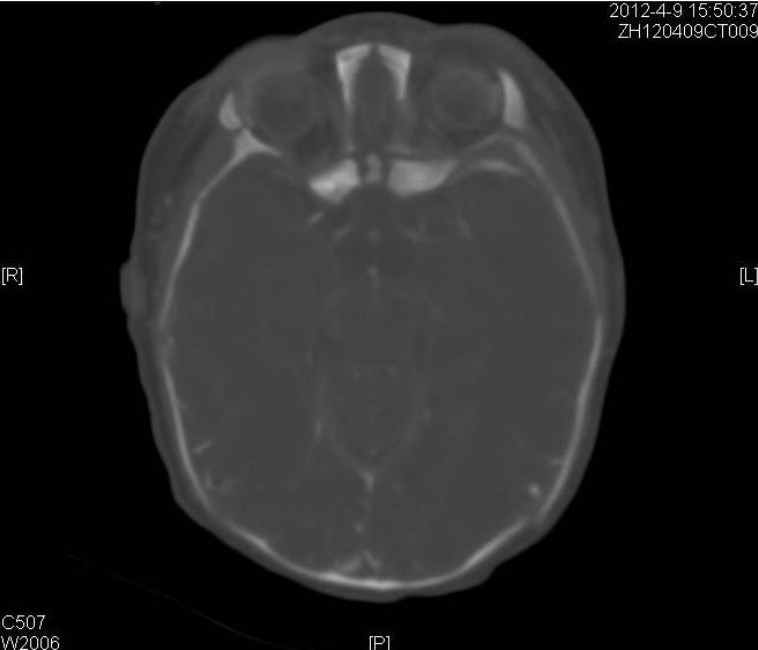
Figure 2: CT scan showing microphthalmia

**Figure:**
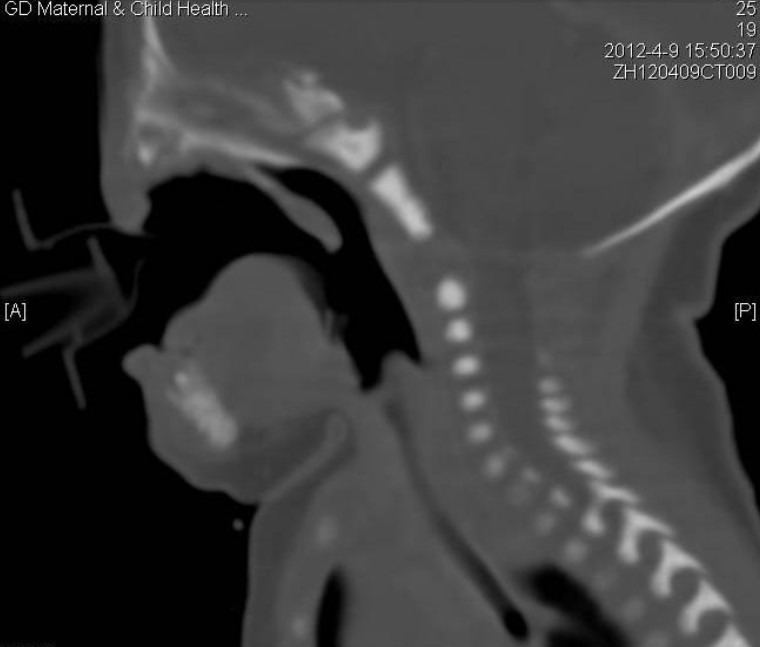
Figure 3: CT scan showing high arched palate, nostril atresia, the hypoplasia of bilateral maxillary sinus and ethmoid sinus

The embryogenesis of nose occurs between the third to eighth week of life. Two fast growing ridges (nasal placodes) develop in the fifth week, forming the medial and lateral nasal swellings and giving rise to the nasal pits between the swellings. The medial swellings fuse to form the nasal septum. Cells within the nasal pits continue to migrate posteriorly to form the primitive nasal cavities, which are separated from the buccal cavity by the rudimentary palatal shelves. It is postulated that lack of development of the nose results from medial failure and lateral nasal process growth, but it is also possible that overgrowth and premature fusion of the nasal medial process result in the formation of an atretic plate [1, 3, 5]. Several genes involved in the nose and facial development have been recognized, however, no consistent gene mutations have been discovered; therefore, genetic testing is not yet available. Only few cases had abnormal chromosomal analysis [6]. Our case also had normal chromosomal analysis like majority of patients with congenital arhinia.

The clinical consequences of congenital arhinia are severe airway obstruction and inability to feed. The nursing care for children is especially important because the infant can’t eat and breathe at the same time without the normal nose function which will result in respiratory distress. A surgically created nasal airway or a tracheostomy tube is an important part of early management. The surgical correction of nasal airway during the neonatal period has been advocated to reduce the potential of dependency on mouth breathing or tracheostomy. Feeding difficulties secondary to impaired simultaneous sucking and breathing may be overcome by placement of an orogastric tube or a gastrostomy tube [1-6]. 

The reconstruction of arhinia is very complex and it should performed by a multidisciplinary team which includes ENT and plastic surgeons, and prosthodontists. The arhinia reconstruction includes reconstruction of the nasal cavity and reconstruction of the external nose. Most authors agree that reconstruction can be delayed at least until preschool years when facial development is nearly completed [7, 8]

## Footnotes

**Source of Support:** Nil

**Conflict of Interest:** None

